# Estimating the population size of gay, bisexual, and other men who have sex with men in four major provinces in Canada: A descriptive study using data from a population-based survey

**DOI:** 10.1371/journal.pone.0330853

**Published:** 2025-09-11

**Authors:** Felipe Tavares Duailibe, David Moore, Mark Hull, Viviane D. Lima

**Affiliations:** 1 University of British Columbia, Department of Medicine, Vancouver, British Columbia, Canada; 2 British Columbia Centre for Excellence in HIV/AIDS, Vancouver, British Columbia, Canada; University of Technology Sydney, AUSTRALIA

## Abstract

**Background:**

Estimating the size of key populations is critical for effective research and policy development. We estimated the population size of gay, bisexual, and other men who have sex with men (GBM) based on different definitions and compared the demographic composition of the GBM and non-GBM populations in Canada.

**Methods:**

This descriptive study used data from the 2015–2016 and 2019–2020 Canadian Community Health Survey (CCHS) cycles. We selected men aged 18–64 years who had valid responses to the sexual identity and sexual behaviour contents. We explored different combinations of the survey questions to estimate the size of the GBM population in Canada and conducted a separate analysis for Canada’s four most populous provinces, comparing sociodemographic characteristics.

**Results:**

Using a definition of GBM combining sexual identity and behaviour (i.e., men who identify as gay or bisexual or who had sex with men in the last 12 months), the weighted proportion of GBM in the 2015–2016 cycle was 2.7% (95% Confidence Interval (CI) 1.9%–3.4%) in Alberta, 3.5% (95% CI 2.7%–4.4%) in British Columbia, 4.1% (95% CI 3.2%–4.9%) in Ontario, and 4.8% (95% CI 4.0%–5.7%) in Quebec. In the 2019–2020 cycle, the weighted proportion of GBM (i.e., men who identify as gay, bisexual or pansexual, or who had sex with men in the last 12 months) was 4.4% (95% CI 3.3%–5.4%) in British Columbia and 4.7% (95% CI 3.9%–5.4%) in Ontario. Overall, compared to non-GBM, GBM were more likely to be single/never married, have an annual household income of less than $30,000, live in medium and large population centres and have lower mean age.

**Conclusion:**

Our estimates showed sexual orientation discordance in Canada. Our findings also suggested that the GBM population might be increasing over time.

## Introduction

Understanding the sociodemographic characteristics of sexual minority populations is essential for addressing disparities and informing policies that promote equity and inclusion [1]. Gay, bisexual, and other men who have sex with men (GBM) represent a key demographic group with unique lived experiences and challenges, making accurate population size estimation critical for effective research and policy development [2]. The legal and societal landscape for GBM in Canada has undergone significant transformation over the last several decades [3]. Homosexuality was decriminalized in Canada in 1969 [3]. Since then, further progress has included the addition of sexual orientation as a prohibited ground for discrimination under human rights legislation and the legalization of same-sex marriage in 2005 [4]. However, challenges such as residual stigma and regional disparities in attitudes toward sexual minorities persist [5], potentially influencing the accuracy of population estimates. Therefore, from a public health perspective, designing, developing, and implementing appropriate prevention, care, and treatment interventions targeting this key population is challenging due to the lack of reliable and representative data on its population size [6].

Given the necessity of determining the underlying population size of individuals who might be more at risk of specific health problems, different methods for estimating the size of the GBM population have been used over the years. These include methodologies such as surveys, capture-recapture and triangulation of multiple methods [6–10]. Each approach has its strengths and weaknesses, and there is no gold standard method [11].

In this context, the Canadian Community Health Survey (CCHS), a national survey administered by Statistics Canada, collects information related to health status, healthcare utilization, and determinants of health representative of 98% of Canada’s population (aged 12 years and over) that can help us estimate the size of the GBM population [12]. From 2003 to 2014, CCHS only included a single question regarding self-reported sexual identity (i.e., *Do you consider yourself to be...? Ans: Heterosexual/Homosexual – that is, lesbian or gay/Bisexual)*, which is one of the three dimensions of sexual orientation (i.e., sexual identity, behaviour, and attraction) [13–15]. However, from a public health perspective, creating estimates of this key population based on the other dimensions of sexual orientation is critical, given that there are discordances among the dimensions of sexual orientation, and the characteristics of each dimension can influence health outcomes [16,17]. For instance, men can identify as heterosexual, yet feel attracted to, and have sexual encounters with other men [18]. At the same time, men can identify as gay, feel attracted to men, and never have sexual encounters with other men [13]. Previous studies that have used CCHS data to develop estimates of the gay and bisexual populations in Canada have focused on sexual identity [14,15]. Therefore, these estimates based on only sexual identity may not accurately capture the population that might be the target for HIV pre-exposure prophylaxis programs (PrEP) or more at risk of sexually transmitted infections (STIs), and a sexual behaviour-based estimate would offer a more precise estimate of men who have sex with men [13].

In 2015, Statistics Canada expanded the CCHS to include specific questions related to sexual behaviour. Since 2015, it is, therefore, now possible to use sexual identity and sexual behaviour questions separately or in combination to estimate the size of the GBM population. In addition, the sociodemographic characteristics of CCHS’s participants can help identify factors influencing GBM’s population size estimates across Canada. In this study, we estimated the population size of GBM using different definitions and data from different CCHS cycles and compared the demographic composition of the GBM and non-GBM populations in Canada.

## Methods

### Data source

Data were obtained from Statistics Canada’s CCHS. The CCHS is a national, cross-sectional survey that collects information on the participants’ relevant sociodemographic and health-related characteristics [12]. The survey is administered to a representative sample of the Canadian population 12 years of age and over, except for persons living on reserves and other Indigenous settlements in the provinces, institutionalized populations, children aged 12–17 that are living in foster care, and persons living in the Quebec health regions of Région du Nunavik and Région des Terres-Cries-de-la-Baie-James [12]. Data is collected using computer-assisted in-person and telephone interviews [12]. The CCHS produces an annual microdata file, which is a file combining two years of data. To provide reliable representative estimates, the two-year targeted sample size is 130,000 respondents, corresponding to 65,000 each year [19]. For all provinces, the data collected in a single year is representative of that setting, except for the territories, given that only half of the communities are visited annually [19]. Therefore, the data is only representative of the territories every two years. The territories refer to the three northern regions of Canada: Yukon, Northwest Territories, and Nunavut [20]. These territories differ from provinces in governance and political structure [20]. A complete description of the complex CCHS design can be found elsewhere [12]. To keep the definitions consistent and comparable across the years, we used data from the CCHS 2015–2016 and 2019–2020 cycles, given that the CCHS 2017–2018 cycle did not include the sexual behaviour content.

### Study sample and design

The design of this study is cross-sectional. For this analysis, we selected men with valid responses to sexual identity and sexual behaviour content. Due to ethics restrictions, we only included men aged 18–64 years. We defined sexual orientation by using the specific questions in the survey that comprised sexual identity and behaviour. For the 2015–2016 cycle, we used combinations of the following survey items:

- Is [Respondent name] male or female? Ans: Male/Female
- Do you consider yourself to be...? Ans: Heterosexual/Homosexual – that is, lesbian or gay/Bisexual;
- During your lifetime, have you had sex with...? Ans: Males/Females/Both;
- In the past 12 months, have you had sex with a male? Ans: Yes/No.

For the 2019–2020 cycle, we combined the following survey items:

- What is your gender? Ans: Male/Female/Or please specify;
- What is your sexual orientation? Ans: Heterosexual/Gay or lesbian/ Bisexual or pansexual/Sexual orientation, not specified elsewhere;
- In the past 12 months, have you had sex with a male? Ans: Yes/No.

We created three definitions for our population based on the combination of these questions: Definition 1, a sexual identity definition of “Men who identify as gay or bisexual”; Definition 2: a sexual behaviour definition of “Men who had sex with men in the last 12 months”; and Definition 3, a combination of sexual identity and behaviour “Men who identify as gay or bisexual OR who had sex with men in the last 12 months”. Since the 2019–2020 cycle changed the sexual identity question to include a pansexual category, Definition 3 for 2019–2020 is “Men who identify as gay, bisexual or pansexual OR who had sex with men in the last 12 months”. It is important to note that although in the 2019–2020 cycle CCHS asked about gender identity, the answers were still “male/female/or please specify”. We defined non-GBM as men who identified as heterosexual and who did not have sex with men in the last 12 months. We estimated the size of the GBM population based on the three definitions.

We used data from all of Canada for the 2015–2016 cycle and conducted a separate analysis for Canada’s four most populous provinces (i.e., Ontario, Quebec, British Columbia and Alberta). In the 2019–2020 cycle, sexual behaviour was included in the survey as optional content, in which provinces and territories included specific questions to address their public health priorities [12]. Therefore, for the 2019–2020 cycle, we included the provinces that opted for the sexual behaviour content (i.e., Ontario, British Columbia, Manitoba, Newfoundland, Labrador and Prince Edward Island), which includes the questions regarding having sex with men in the previous 12 months, and conducted a separate analysis for Ontario and British Columbia. The provinces that comprised this second analysis were selected based on sample size. The less populous provinces had fewer respondents, resulting in a small unweighted sample size and unreliable estimates.

The demographic characteristics presented in the second part of the analysis were marital status, household income and population centres or rural area status. Household income data information was imputed, where missing values resulting from the respondent’s refusal or lack of knowledge of household income were replaced using various techniques, including a nearest neighbour imputation approach based on a modelled household income [19]. The variable “population centres or rural area status” was comprised of four categories: rural area (less than 1,000); small population centre (1,000–29,999); medium population centre (30 000–99,999); and large urban population centre (100,000 or greater). Given the small sample size of GBM living in rural areas in some provinces, we grouped the variable into two categories: men living in areas with less than 30,000 (i.e., rural areas and small population centres) and 30,000 people or more (i.e., medium and large urban population centres) [21].

### Statistical analysis

We used a set of 500 bootstrap weights to estimate uncertainty, accounting for household and person-level non-response and calculated the sampling weight of each respondent [12]. We also incorporated sampling weights into the analysis so that the CCHS estimates represent our covered population at the national, provincial and regional levels [12]. CCHS attributes a survey weight to each respondent included in the final sample, corresponding to the number of persons in the entire population represented by the respondent [12]. A detailed description of the CCHS’s weights can be found elsewhere [12]. In addition, we calculated coefficients of variation and confidence intervals to determine the quality of the estimates regarding sampling variability. We used the chi-squared test to compare the distribution of sociodemographic characteristics of GBM versus non-GBM. All the descriptive analyses were performed using SAS 9.4 using survey procedures, and the significance level was set at 5%.

### Ethics approval

A Microdata Research Contract between the researchers and Statistics Canada was signed, and security clearance was confirmed, granting access to the Research Data Centre to conduct these analyses. Data available at the Research Data Centre were fully anonymized prior to our access. Therefore, informed consent was not obtained for this study. We first accessed the data for research purposes on 13/04/2021. Ethics approval was acquired from the Providence Health Care Research Ethics Board (H18-00949-A012).

## Results

Table 1 shows the weighted estimated number of GBM identified in Canada based on different definitions derived from the survey responses, including sexual identity, sexual behaviour and sexual identity and behaviour combined (reported here as Definitions 1–3, respectively). Considering Definition 1, based on sexual identity only, the proportion of GBM increased from 2015–2016 to 2019–2020 from 3.1% (95% CI 2.7–3.4) in the 2015–2016 cycle to 3.9% (95% CI 3.3–4.4) in 2019–2020.

**Table 1:** Distribution of the Gay, Bisexual and other Men who have Sex with Men population aged 18-64 years in Canada based on the 2015–2016 and 2019–2020 cycles of the Canadian Community Health Survey by differing sexual identity definition.

	Definition	Weighted percentage	95% CI weighted percentage
	**CCHS 2015–2016**		
1:	Men who identify as gay or bisexual	3.1	2.7–3.4
2:	Men who had sex with men in the last 12 months	2.4	2.0–2.7
3:	Men who identify as gay or bisexual OR who had sex with men in the last 12 months	3.8	3.4–4.2
	**CCHS 2019–2020**		
1:	Men who identify as gay, bisexual or pansexual	3.9	3.3–4.4
2:	Men who had sex with men in the last 12 months	2.7	2.3–3.2
3:	Men who identify as gay, bisexual or pansexual OR who had sex with men in the last 12 months	4.5	3.9–5.1

Note: CCHS = Canadian Community Health Survey. CI = Confidence interval. CCHS cycle 2015–2016 includes respondents from all provinces and territories. CCHS 2019–2020 includes Ontario, British Columbia, Manitoba, Newfoundland and Labrador and Prince Edward Island.

In the 2015–2016 cycle, restricting the analysis to Ontario, Quebec, British Columbia and Alberta and using Definition 3, 4.0% (95% CI 3.5–4.4) of the male respondents were estimated to be GBM (Table 2). Quebec was the province with the largest GBM population estimate (4.8%, 95% CI 4.0–5.7), and Alberta had the smallest estimate (2.7%, 95% CI 1.9–3.4) (Table 2).

**Table 2:** Estimation of the population size of Gay, Bisexual and other Men who have sex with Men aged 18–64 years in Alberta, British Columbia, Ontario and Quebec.

	Weighted percentage; 95% Confidence Interval of weighted percentage (Weighted frequency)				
CCHS 2015–2016	Alberta	British Columbia	Ontario	Quebec	Total
**Men who identify as gay or bisexual, or who had sex with men in the last 12 months**					
No	97.3; 96.5–98.1 (1,209,075)	96.5; 95.6–97.3 (1,184,003)	95.9; 95.0–96.8 (3,400,994)	95.2; 94.3–96.0 (2,252,322)	96.0; 95.5–96.4 (8,046 ,392)
Yes	2.7; 1.9–3.4 (33,281)	3.5; 2.7–4.4 (43,328)	4.1; 3.2–4.9 (144,260)	4.8; 4.0–5.7 (114,585)	4.0; 3.5–4.4 (335,455)
**CCHS 2019–2020**					
**Men who identify as gay, bisexual or pansexual, or who** **had sex with men in the last 12 months**					
No	*	95.6; 94.6–96.7 (1,282,400)	95.3; 94.6–96.1 (3,767,818)	*	95.4; 94.8–96.0 (5,050,218)
Yes		4.4; 3.3–5.4 (58,497)	4.7; 3.9–5.4 (185,057)		4.6; 4.0–5.2 (243,554)

Note: *Province did not opt for the sexual behaviour content in the 2019–2020 cycle; therefore, estimates were not calculated. CCHS = Canadian Community Health Survey.

Table 3 shows the demographic composition of the male population in Alberta, British Columbia, Ontario and Quebec and compares GBM vs. non-GBM in 2015–2016. In the 2015–2016 cycle, compared to non-GBM, GBM were more likely to be single/never married (54.9% vs. 26.8%, p-value < 0.001), have an annual household income less than 30,000 dollars (14.3% vs. 9.1%, p-value < 0.001), live in medium and large population centres (84.5% vs. 74.2%, p-value < 0.001), and have lower mean age (38.8 years vs. 42.0 years, p-value <0.001) (Table 3).

**Table 3:** Demographic characteristics of the male population aged 18–64 years in Alberta, British Columbia, Ontario and Quebec based on the 2015–2016 Canadian Community Health Survey.

	Demographic characteristics	Weighted percentage; 95% Confidence Interval of weighted percentage (Weighted frequency)					
	**CCHS 2015–2016**	**Alberta**	**British Columbia**	**Ontario**	**Quebec**	**Total**	**p-value**
	**Marital status**						< 0.001
Non-GBM	Single, never married	23.6; 21.6–25.6 (284,905)	27.1; 25.2–28.9 (319,662)	26.8; 25.3–8.30 (910,965)	28.3; 26.7–29.7 (636,958)	26.8; 25.9–27.7 (2,152,491)	
	Married/common–law	70.2; 67.9–72.3 (846,290)	66.1; 64.1–68.1 (780,945)	66.3; 64.5–68.0 (2,249,944)	65.4; 63.7–67.1 (1,474,258)	66.6; 65.5–67.6 (5,351,437)	
	Divorced, separated, widowed	6.2; 5.3–7.0 (74,555)	6.8; 5.7–7.7 (80,028)	6.9; 6.00–7.72 (232,891)	6.3; 5.4–7.0 (141,105)	6.6; 6.07–7.08 (528,580)	
GBM	Single, never married	52.9; 38.6–67.3 (17,637)	52.0; 40.5–63.5 (22,534)	59.0; 48.9–69.0 (84,833)	51.4; 43.1–9.6 (58,903)	54.9; 49.3–60.5 (183,907)	
	Married/common–law	42.9; 28.4–57.4 (14,297)	42.7; 30.8–54.5 (18,508)	34.7; 24.7–44.7 (49,980)	42.5; 34.0–50.8 (48,639)	39.2; 33.6–44.8 (131,423)	
	Divorced, separated, widowed	E	E	6.3; 3.2–9.3 (9,047)C	6.1; 2.9–9.4 (7 044 )C	5.9; 3.9–7.8 (19,725)	
	**Household income**						
Non-GBM	<30000	4.7; 3.7–5.6 (56,749)	9.3; 8.0–10.6 (110,312)	9.2; 8.0–10.36 (312,958)	11.3; 10.1–12.4 (254,678)	9.1; 8.45–9.8 (734,697)	<0.001
	≥30000	95.3; 94.4–96.2 (1,152,326)	90.7; 89.3–91.9 (1,073,691)	90.8; 89.6–91.9 (3,088,035)	88.7; 87.5–89.8 (1,997,643)	90.9; 90.1–91.5 (7,311,696)	
GBM	<30000	E	13.0; 6.05–19.8 (5,616)C	14.6; 8.6–20.5 (21,056)C	17.4; 11.7–23.0 (19,932)C	14.3; 10.9–17.6 (47,928)	
	≥30000	96.0; 91.8–100 (31,957)	87.0; 80.1–93.9 (37,712)	85.4; 79.5–91.3 (123,204)	82.6; 76.9–88.2 (94,653)	85.7; 82.3–89.1 (287,527)	
	**Population centre status**						
Non-GBM	Rural area and small population centres	29.2; 26.9–31.4 (352,876)	24.4; 22.3–26.5 (289,338)	22.4; 21.0–23.8 (762,866)	29.8; 27.6–31.8 (670,280)	25.8; 24.8–26.7 (2,075,360)	<0.001
	Medium and large population centres	70.8; 68.5–73.0 (856,199)	75.6; 73.4–77.6 (894,665)	77.6; 76.1–78.9 (2,638,128)	70.2; 68.1–72.3 (1,582,041)	74.2; 73.2–75.1 (5,971,033)	
GBM	Rural area and small population centres	17.5; 7.1–27.8 (5,823)D	17.4; 8.9–25.7 (7,518)C	10.4; 6.8–13.9 (14,933)C	20.8; 14.9–26.5 (23,796)	15.5; 12.4–18.5 (52,070)	
	Medium and large population centres	82.5; 72.1–92.8 (27,459)	82.6; 74.2–91.0 (35,810)	89.6; 86.1–93.2 (129,327)	79.2; 73.4–85.0 (90,789)	84.5; 81.4–87.5 (283,385)	
	Continuous demographic characteristic						
	**Mean age**						
Non-GBM		40.8 (40.4–41.1)	42.2 (41.8–42.5)	42.0 (41.7–42.3)	42.4 (42.1–42.7)	42.0 (41.8–42.1)	<0.001
GBM		37.2 (33.6–40.7)	39.6 (36.8–42.5)	38.1 (35.1–41.0)	39.9 (37.7–42.1)	38.8 (37.2–40.4)	

Note: CCHS = Canadian Community Health Survey. GBM = Men who identify as gay, bisexual OR who had sex with men in the last 12 months. Non-GBM = Men who identified as heterosexual but did not have sex with men in the last 12 months. C = estimates of marginal acceptability due to high sampling variability (Coefficient of Variance 0.15–0.25). D = estimates of marginal acceptability due to high sampling variability (Coefficient of Variance 0.25–0.35). E = estimates of unacceptable quality due to high sampling variability (Coefficient of Variance > 0.35).

In the 2019–2020 cycle, considering Definition 3 for British Columbia and Ontario, 4.6% (95% CI 4.0–5.2) of the respondents were estimated to be GBM (Table 2). Table 4 shows the demographic composition of the population in British Columbia and Ontario and compares GBM vs. non-GBM in 2019–2020. Compared to non-GBM, GBM were more likely to be single/never married (55.4% vs. 26.0%, p-value < 0.001), have an annual household income of less than 30,000 dollars (11.7% vs. 6.9%, p-value < 0.001), live in medium and large population centres (87.3% vs. 76.5%, p-value <0.001), and have lower mean age (38.3 vs. 42.3 years, p-value= < 0.001) (Table 4).

**Table 4:** Demographic characteristics of men aged 18–64 years in British Columbia and Ontario based on the 2019–2020 Canadian Community Health Survey.

	Demographic characteristics	Weighted percentage; 95% Confidence Interval of weighted percentage (Weighted frequency)			
	CCHS 2019–2020	British Columbia	Ontario	Total	p–value
	**Marital status**				
Non-GBM	Single, never married	23.7; 21.0–26.7 (302,751)	27.1; 25.5–28.7 (1,019,784)	26.2; 24.8–27.7 (1,322,535)	<0.001
	Married/common–law	70.5; 67.6–73.4 (901,051)	67.5; 65.7–69.2 (2,540,926)	68.2; 66.7–69.8 (3,441,976)	
	Divorced, separated, widowed	5.8; 4.8–6.9 (74,597)	5.4; 4.8–6.1 (205,066)	5.5; 5.0–6.1 (279,663)	
GBM	Single, never married	54.9; 42.8–67.0 (32,108)	55.5; 46.8–64.3 (102,607)	55.4; 48.2–62.5 (134715)	
	Married/common–law	40.1; 27.7–52.5 (23,461)C	39.4; 30.9–47.9 (72,744)	39.6; 32.6–46.5 (96205)	
	Divorced, separated, widowed	E	5.1; 0.9–9.2 (9,397)D	5.1; 1.8–8.3 (12,325)D	
	**Household income**				
Non-GBM	<30000	6.5; 5.2–7.8 (83,240)	6.9; 6.0–7.9 (260,200)	6.8; 6.0–7.6 (343,440)	<0.001
	≥30000	93.5; 92.2–94.8 (1,199,160)	93.1; 92.1–94.0 (3,507,618)	93.2; 92.4–94.0 (4,706,778)	
GBM	<30000	15.6; 6.8–24.4 (9,122)D	10.4; 5.2–15.7 (19,303)D	11.7; 7.1–16.3 (28,425)C	
	≥30000	84.4; 75.6–93.2 (49,375)	89.6; 84.3–94.8 (165,754)	88.3; 83.7–92.9 (215,129)	
	**Population centre status**				
Non-GBM	Rural area and small population centres	21.3; 19.2–23.4 (272,919)	23.6; 22.3–24.8 (887,886)	23.0; 21.9–24.1 (1,160,805)	<0.001
	Medium and large population centres	78.7; 76.6–80.8 (1 009 ,481)	75.2; 75.2–77.7 (2,879,932)	77.0; 75.9–78.1 (3,889,413)	
GBM	Rural area and small population centres	9.2; 4.2–14.2 (5,410)D	13.8; 8.7–18.9 (25,534)C	12.7; 8.7–16.7 (30,944)C	
	Medium and large population centres	90.8; 85.8–95.8 (53,086)	86.2; 81.1–91.3 (159,523)	87.3; 83.3–91.3 (212,610)	
	Continuous demographic characteristic	Weighted mean (95% Confidence Interval of the weighted mean)			
	**Mean age**				
Non-GBM		42.5 (42.0–43.0)	42.3 (42.0–42.6)	42.3 (42.1–42.6)	<0.001
GBM		38.9 (35.8–42.0)	38.1 (35.6–40.6)	37.2 (36.2–40.3)	

Note: CCHS = Canadian Community Health Survey. GBM = Men who identify as gay, bisexual or pansexual OR who had sex with men in the last 12 months. Non-GBM = Men who identified as heterosexual but did not have sex with men in the last 12 months. C = estimates of marginal acceptability due to high sampling variability (Coefficient of Variance 0.15–0.25). D = estimates of marginal acceptability due to high sampling variability (Coefficient of Variance 0.25–0.35. E = estimates of unacceptable quality due to high sampling variability (Coefficient of Variance > 0.35)

Comparing Definition 3 estimates, which combines sexual identity with sexual behaviour British Columbia increased from 3.5% (95% CI 2.7–4.4) in 2015–2016 to 4.4% (95% CI 3.3–5.4) in 2019–2020. However, the confidence intervals overlap (Table 2).

## Discussion

Our estimates, based on the CCHS, found that relying on only one domain of sexual orientation might not be sufficient to capture the entire GBM population in Canada. We saw that men who identify as gay or bisexual OR who had sex with men in the last 12 months corresponded to 3.8% of the male population in Canada in 2015–2016. On the other hand, using a purely behaviour-based definition (i.e., men who had sex with men in the last 12 months), we identified that 2.4% of the male Canadian population were GBM in 2015–2016. In 2019–2020, men who identify as gay, bisexual or pansexual OR who had sex with men in the last 12 months, comprised 4.5% of the male population, and men who had sex with men in the last 12 months corresponded to 2.7%. In Ontario, Quebec, British Columbia and Alberta, in 2015–2016, 4.0% of the respondents were estimated to be men who identify as gay or bisexual OR who had sex with men in the last 12 months. In British Columbia and Ontario, 4.6% of the respondents in 2019–2020 were estimated to be men who identify as gay, bisexual or pansexual OR who had sex with men in the last 12 months. Overall, GBM in these two periods were predominantly single/never married, had an annual household income of more than 30,000 dollars and lived in medium and large population centres.

Studies have demonstrated sexual orientation discordance in the United States, Britain and Canada [13,17, 22]. In our study, we showed different estimates using sexual identity and behaviour separately and combined. These estimates should be used carefully according to their specific needs and assumptions made for the targeted population, knowing whether sexual identity, behaviour or both combined would be more appropriate. For instance, Definition 1 (identity-based) is more appropriate when referring to people more likely to experience stigma and discrimination [13]. Definition 2 (behaviour-based) should be used when referring to the population more at risk of HIV and other STIs [13]. And finally, Definition 3 (identity and behaviour) should be used when the health outcome is more present in both groups, as seen in mental health disorders such as anxiety and depression [23–25].

Our findings also complement previous studies estimating the size of the GBM population in Canada. In 2016, the British Columbia Center for Disease Control estimated the men who have sex with men population in British Columbia to be 2.6% of the male population using data from the 2013–2014 CCHS cycle, adjusting for under-reporting [26]. A similar estimate was found in a study conducted in Vancouver (2.9%), which used multiple datasets and methods, including estimates from the CCHS, Wisdom of the Crowds, and the multiplier method [8]. As previously mentioned, CCHS only included the sexual behaviour question we used to create our GBM population estimates in 2015. Therefore, the higher estimate of the GBM population in British Columbia observed in our study might be due to the capture of more GBM using the combined identity-behaviour definition. A more recent study combining data from two surveys (Sex Now 2019 and CCHS 2017, 2018 and 2019) [27] created GBM estimates using sexual identity and behaviour-based questions. This study produced estimates for each data source, adjusted for nondisclosure of sexual identity, and then averaged the estimates [28]. They found that 4.1%, 6.6% and 4.8% of the adult male population identified as gay and bisexual in Ontario, Toronto and Ottawa, respectively. In addition, when they used a behaviour-based definition, only including sexually active GBM, the proportion of GBM reduced to 2.8% in Ontario [28]. Comparing our results to the findings of this study, our estimates for Ontario differ in the combined identity-behaviour estimates. Instead of retaining only sexually active GBM, we created an estimate that included both, sexually active (i.e., men who had sex with men in the last 12 months) and men who identified as gay or bisexual (and pansexual in 2019–2020), which might explain the difference in the estimates. Similarly, a national study using Sex Now 2019 and CCHS 2020 estimated the sexual behaviour-based GBM population to be 2.7% and the combined identity and behaviour-based GBM population to be 4.1% of the male population [29]. In this study, the identity-behaviour-based estimate is comparable and similar to ours, given that they combined both populations, not excluding non-sexually active GBM.

We observed an overall increased proportion of GBM from 2015–2016 to2019–2020. Although the confidence intervals overlapped, this could be an indication of the increasing GBM population. We believe that cultural and political changes in Canada might explain those differences. Historically, same-sex practices and relationships globally carry stigma on different levels, being criminalized in at least 76 countries and exposing millions of individuals to the risk of arrest, prosecution, imprisonment and the death penalty [30]. In Canada, same-sex sexual activities were considered a crime punishable by imprisonment before 1969 [3]. Since then, legislation has changed, and, currently, sexual orientation is included in human rights legislation as prohibited grounds for discrimination with same-sex civil marriage, one of the most significant recent progressions in LGBTQ rights (Lesbian, Gay, Bisexual, Transgender and Queer), being legalized in 2005 nationwide [3,4]. It is also important to note that, in 2015, a new policy agenda for LGBTQ legal reform was introduced by the Liberal government, which included the reform of the Criminal Code, a government apology to LGBTQ Canadians for past discrimination, and the expungement of records of past criminal convictions for same-sex behaviour [4]. Consequently, we believe that as more public policies are created and implemented to protect sexual minorities over time, GBM will be more likely to feel comfortable disclosing their sexual orientation on a government national survey; this is likely reflected in the recent estimates.

Emphasizing the possible changes in the willingness to disclose sexual orientation, the proportion of Americans who self-identify as LGBTQ has been increasing over time. Gallup’s (an American analytics and advisory company) latest update on LGBTQ self-identification concluded that the LGBTQ population increased from 3.5% in 2012 to 9.3% in 2024 [31]. One of the main reasons for the observed change in the proportions was that younger generations are more likely to disclose themselves as LGBTQ than older generations [31]. In addition, a study conducted in Canada observed that there were age disparities in the willingness to disclose sexual orientation to a government survey, with older men less likely to reveal it [32]. In our study, we observed a lower mean age of GBM compared to non-GBM. Comparing 2015–2016 to 2019–2020, we found that the mean age of the GBM respondents decreased from 39.6 years in British Columbia and 38.1 years in Ontario, to 37.3 and 37.2 years, respectively, which could contribute to explaining the increase in the more recent GBM estimates.

We also observed that GBM were more likely to live in medium or large urban centres than their non-GBM counterparts. The migration of GBM to large urban centres is a phenomenon observed globally [33–35]. Large cities often have more diverse populations and cultural landscapes with a sense of community belonging and identity affirmation for sexual minorities. Therefore, GBM in large cities can express their sexuality more freely, likely impacting their willingness to disclose their sexual orientation compared to GBM. A qualitative study conducted in the United States among closeted GBM has shown that conservative perceptions of traditional masculine gender norms and unfavourable attitudes toward same-sex relationships in rural areas have influenced them against disclosing their sexual orientation [36]. This finding could also reflect on the GBM population estimates of more conservative or rural provinces in Canada, underestimating the proportion of GBM.

### Limitations

There are some limitations to our findings. Definition 3 did not include men who had sex with men who reported having sex more remotely than in the most recent 12 months. However, retaining the definitions with the inclusion of recent sexual intercourse with another man was more appropriate for our study to express a more contemporary assessment of their sexual behaviour. The sexual identity-based questions differ from the 2015–2016 to2019–2020 cycles, including the “pansexual” identity in 2019–2020. Although this is a minor difference, it is important to mention this limitation to show the evolution of new sexual identity terminology in a nationwide survey. Additionally, the variable “gender” was only included in CCHS for the 2019–2020 cycle. Before that, only the variable “sex at birth” was available. Therefore, we might have missed heterosexual transgender men and transgender men who have sex with men who do not identify as gay, bisexual or pansexual, and might have included transgender women who had sex with men in the 2015–2016 cycle.

In 2019–2020, the estimation of the size of the GBM population using Definition 1 for Canada 2019–2020 did not include all provinces and territories. We intended to estimate the GBM population size for Canada’s four main provinces in 2019–2020. However, in this cycle, the sexual behaviour content was unavailable for all Canadian provinces.

Another crucial limitation when working with CCHS data is the residual disclosure risk, i.e., the potential for individuals’ identities to be indirectly revealed by releasing survey data. As we used close variations of highly specific definitions, the risk of identification impacted my ability to explore the data completely and, consequently, produce results. Since, in this study, unweighted data were stratified by province and used closely related but finely distinguished definitions, we had to restrict the number of sociodemographic characteristics used and the number of possible combinations of CCHS questions regarding sexual orientation to avoid the risk of unintentionally re-identifying individuals who appeared in small categories. In addition, the number of data points in some sociodemographic characteristics cells was very small, resulting in high coefficients of variance and producing unreliable estimates. We were unable to report any results for these cells. Finally, our estimates could be underestimated. As sexual orientation and sexual behaviour might be seen as sensitive subjects to some people, participants might have given answers that they thought were more acceptable and less stigmatizing. Therefore, when asked if they were GBM, men might feel pressured to say “no”, not to disclose their sexual orientation, or to omit their actual sexual behaviour.

## Conclusions

In conclusion, this study used sexual identity and behaviour to estimate the size of the GBM population based on a national population-based survey. We found that, in 2015–2016, the size of the GBM population, using the hybrid definition combining sexual identity and behaviour (i.e., men who identify as gay or bisexual or who had sex with men in the last 12 months), corresponded to 4.0% of the male population in Alberta, British Columbia, Ontario and Quebec, and, in 2019–2020, the GBM population (i.e., men who identify as gay, bisexual or pansexual, or who had sex with men in the last 12 months) corresponded to 4.6% of the male respondents. We also found that compared to non-GBM, GBM were more likely to be single/never married, have an annual household income of less than $30,000, live in medium and large population centres, and have lower mean age. Additionally, we observed an increase in the GBM population comparing the 2015–2016 to the 2019–2020 CCHS cycle, possibly due to people being more comfortable in disclosing their sexual orientation and behaviour.

Our estimates are valuable to public health practitioners, policymakers and researchers since we can now provide denominators to estimate GBM-specific disease rates, such as HIV and other sexually transmitted infections, and evaluate rates of anxiety, depression and other psychological problems specifically in GBM. In addition, our behaviour-based definition could serve as a denominator to forecast GBM that need PrEP and evaluate PrEP uptake. It is important to emphasize that sexual orientation is a multidimensional concept and that there is discordance across the domains. Therefore, the estimations presented here, based on different domains of sexual orientation, should be used cautiously, with a relevant and clear rationale for their use.
